# Protective Effect of Citrulline on the Hearts of Rats with Sepsis Induced by Cecal Ligation and Puncture

**DOI:** 10.1155/2018/2574501

**Published:** 2018-10-18

**Authors:** Ji-qiu Zhou, Xiong Xu, Wei-wei Zhen, Yu-long Luo, Bin Cai, Sen Zhang

**Affiliations:** Department of Colorectal Surgery, First Affiliated Hospital of Guangxi Medical University, Nanning, Guangxi 530021, China

## Abstract

**Purpose:**

To investigate the protective effect of citrulline (Cit) on the hearts of rats with sepsis.

**Methods:**

Wistar rats were divided into the normal, sham-operated, CLP, Cit, and CLP+Cit groups. Routine blood tests were performed, and the blood biochemical indexes were measured. Pathological changes in the cardiac tissues were observed. The levels of NO and iNOS in blood and SOD activity and MDA levels in the heart were measured.

**Results:**

Less inflammatory cell infiltration of the myocardial fibers and significantly decreased white blood cell count, absolute neutrophil count, neutrophil percentage, CK, HBDH, and NO (all* P*<0.05) were detected in the CLP+Cit group compared with the CLP group. In addition, SOD activity and MDA levels in heart tissues were, respectively, higher and lower in the CLP+Cit group than in the CLP group (both* P*<0.05).

**Conclusions:**

Cit reduces pathological damage in the heart and enhances the heart's antioxidant capacity, thereby protecting cardiomyocytes.

## 1. Introduction

Sepsis may progress to systemic inflammatory response syndrome (SIRS), severe sepsis, septic shock, and multiple-organ dysfunction syndrome (MODS), which has been defined as SIRS caused by previous infection and trauma [1]. In 2015, the Society of Critical Care Medicine (SCCM) redefined sepsis as organ dysfunction and complications caused by uncontrolled inflammation. A Sepsis-related Organ Failure Assessment (SOFA) score ≥2 indicates sepsis. Septic shock is defined as a specific type of sepsis, and the risk of death from septic shock is significantly higher than that from simple sepsis [2]. Cardiovascular disease, cancer, and sepsis are the three most common fatal diseases in the United States [3]. Moreover, with the aging of the population and increases in cancer incidence and invasive medical treatments, the incidence of sepsis is gradually increasing.

Cardiac dysfunction and myocardial infarction caused by sepsis-induced myocardial injury are common causes of death in sepsis patients. Prevention and control of myocardial injury in sepsis patients can effectively reduce sepsis patient mortality. At present, the main reasons for organ damage caused by sepsis include inflammatory cytokines and oxidative stress injury. An increase in cardiomyocyte apoptosis can increase iNOS and NADPH oxidase expression as well as NO and superoxide production, thereby inducing oxidative stress and other damage [4–6]. Antioxidant enzymes are commonly assessed to determine the extent of cell damage after an oxidative stress response. Therefore, we measured superoxide dismutase (SOD), which is more representative than other biochemicals, to reflect the extent of oxidative stress injury. Additionally, we measured malondialdehyde (MDA), a commonly used index that reflects lipid peroxidation in the body, to indirectly determine the extent of cell damage.

Arginine (ARG) is a basic amino acid that plays an important role in cell regeneration, wound healing, protein transformation, and immunity [7]. In recent years, several studies of sepsis have found that the plasma level of ARG significantly decreases when sepsis occurs [8]. As the main pathogenic factors that induce sepsis and MODS, lipopolysaccharide (LPS) and endotoxin can activate inflammatory factors at different stages of the development of sepsis in response to increased inducible nitric oxide synthase (iNOS) release, which leads to increased NO synthesis and increased and accelerated ARG consumption [9]. Additionally, increased expression of arginase may occur in sepsis [10].

However, a randomized controlled clinical trial showed that ARG supplementation did not significantly improve the fatality rate in hospitals, incidence of infective complications, and length of hospital stay in the ICU [11]. The main reason is that ARG supplementation leads to excess NO production via iNOS, which increases the cardiovascular burden in septic patients [12].

Citrulline (Cit) is one type of L-*α*-amino acid that does not exist in proteins. ARG can be broken down into Cit by iNOS [13]. The molecular structure of Cit is similar to that of ARG; thus, Cit can competitively inhibit the active site of iNOS, thereby negatively regulating the production of NO. This process is called the Arg-Cit cycle or Cit-NO cycle [14].

Due to the potential regulatory role of Cit in the Cit-NO cycle, this study established a rat model of sepsis by cecal ligation and puncture (CLP) and supplemented the rats with exogenous Cit to explore the changes in their heart tissue pathology, blood cell count, heart function, and antioxidant capacity. We aimed to develop a new method for the prevention of myocardial injury and the protection of cardiac function in sepsis.

## 2. Materials and Methods

### 2.1. Ethics Approval

This study was conducted in strict accordance with the guidelines for the Care and Use of Laboratory Animals and approved by the Ministry of Science and Technology of China. The experimental procedures were approved by the Ethics Committee of the Institute of Guangxi Medical University (Approval no. 2015 (K-Y-E-005)).

### 2.2. Choice of Rats

A total of 120 specified pathogen-free (SPF) male rats aged 7 to 10 weeks and of 250~300 g in body weight were provided by the Experimental Animal Center of Guangxi Medical University. All rats adapted to the environment in the SPF animal facility over a period of one week. Each rat was reared in a single cage (485×350×200 mm). The ambient temperature was maintained at 22~25°C, and the air humidity was set at 45~55%. All animals were subjected to a normal circadian rhythm (12 h:12 h light-dark cycle). The rats were allowed to eat and drink ad libitum. The health of the animals was specifically monitored and recorded once every 6 h.

### 2.3. Grouping of Rats

Rats were divided into five groups according to a randomizing number table as follows: normal group (n=8), sham group (n=8), Cit group (n=8), CLP group (n=52), and CLP+Cit group (n=52). The CLP group and the CLP+Cit group were further separated into the following three subgroups: 6 h, 12 h, and 24 h, according to the time of sacrifice after CLP surgery. The number of rats in each group was as follows: CLP 6 h group (n=12), CLP 12 h group (n=16), CLP 24 h group (n=24), CLP+Cit 6-h group (n=12), CLP+Cit 12-h group (n=16), and CLP+Cit 24-h group (n=24).

### 2.4. CLP Rat Model

The Wistar rats were weighed and numbered, and some were anesthetized by intraperitoneal injection with 10% chloral hydrate at a dose of 0.35 ml/100 g of body weight.

#### 2.4.1.  

Rats in the normal group did not receive special treatment.

#### 2.4.2.  

The abdomen of each rat in the sham group was opened by a 1.5 to 2 cm incision on the anterior abdominal wall after anesthesia. The incision was sutured after the cecum was slightly compressed.

#### 2.4.3.  

The CLP operation steps were previously reported in the studies by Dejager, Pinheiro, Dejonckheere, and Libert [15] and Toscano, Ganea, and Gamero [16]. After opening the abdominal cavity and carefully separating the mesentery, the cecum of each rat in the CLP and CLP+Cit groups was ligated with a 4-0 silk suture. After gently pushing the cecum contents to the distal end of the cecum, the intestinal wall was then punctured. A small amount of intestinal contents was squeezed from the puncture holes, and the cecum was then placed back in its original location before the incision was sutured. Surgical principles of antisepsis were followed for the above experimental operation.

#### 2.4.4.  

Each rat in the Cit and CLP+Cit groups received L-citrulline (purity ≥99%, RUIBIO, Germany) by intragastric administration for 7 days before any other surgical procedure (dose of 900 mg kg^−1^·d^−1^).

### 2.5. Sample Collection and Processing

The surviving rats were sacrificed at 6, 12, or 24 h after surgery. Blood was collected via the abdominal aorta for analysis and the extraction of serum. Heart tissues were removed and rinsed in 4°C normal saline. After removing the cardiac pericardium and vessels, a portion of the heart tissue from the normal, sham, Cit, CLP, and CLP+Cit groups was fixed in 10% formalin to generate paraffin-embedded sections. Pathological changes in the HE-stained heart tissues were observed using a light microscope. The rest of the tissues were immediately frozen at -80°C for further analysis.

### 2.6. Routine Blood Tests and Serum Tests

A total of 2 ml of anticoagulated blood from each rat was used to determine the leukocyte count, erythrocyte count, hemoglobin concentration, platelet count, absolute neutrophil count, and neutrophil percentage using a routine blood analyzer. A total of 1 ml of serum from each rat was taken to test the level of serum creatine kinase (CK), creatine kinase isoenzyme (CK-MB), lactate dehydrogenase (LDH), and *α*-hydroxybutyrate dehydrogenase (*α*-HBDH) via an automatic biochemical analyzer.

### 2.7. Tests for NO, iNOS, SOD, and MDA

Appropriate amounts of rat serum were used for testing. The NO levels and iNOS activity in the serum were measured using an NO colorimetric assay kit and NOS assay kit, respectively (Nanjing Jiancheng Bioengineering Institute, China). A 10% homogenate of heart tissue was generated using 100 mg of rat heart tissue and 900 *μ*l of normal saline. The homogenate was put in a Heraeus Sepatech centrifuge (2500 rpm) for 10 min at 4°C. The quantity of protein in the supernatant was determined using the Coomassie Brilliant Blue method. The enzymatic activity of SOD and the levels of MDA in heart tissues were evaluated via a microplate reader (SpectraMax Plus 384 MD, USA) and commercial reagent kits (Nanjing Jiancheng Bioengineering Institute, China).

### 2.8. Statistical Analysis

All data were statistically analyzed with SPSS 17.0 software. Tests of normality were performed on all measurement data. Standard values are presented as the means ± standard deviation (SD). Data were analyzed using Student's* t*-test and one-way ANOVA.* P*<0.05 was regarded as statistically significant.

## 3. Results

### 3.1. Survival of the Rats

The survival rate of the sham group was 100% (8/8) at 24 h postoperatively. After 7 days of intragastric administration of Cit, the survival rates of the Cit and CLP+Cit groups were both 100% (8/8 and 52/52, respectively). At the 6-h, 12-h, and 24-h time points after the operation, the survival rates of the CLP group were 75% (9/12), 50% (8/16), and 33.33% (8/24), respectively, and the survival rates of the CLP+Cit group were 83.33% (10/12), 68.75% (11/16), and 66.67% (16/24), respectively. The chi-squared test was used to compare the survival rates between the CLP group and the CLP+Cit group at the same time points. We found that the survival rate of the 24-h CLP+Cit subgroup (66.67%) was significantly higher than that of the 24-h CLP subgroup (33.33%) (*P*=0.042). Additionally, at the 6-h and 12-h postoperative time points, the survival rates were higher in the CLP+Cit group than in the CLP group; however, the difference was not statistically significant (*P*>0.05).

### 3.2. General Condition and Intra-Abdominal Lesions of the Rats

Compared with the normal group, the rats that underwent CLP surgery exhibited different degrees of disease symptoms according to the time of sacrifice. Rats exhibited symptoms of reduced activity, fluffy fur, and reduced food intake at 6 h after CLP surgery. At 12 h after CLP, the rats curled up their bodies and exhibited accelerated heart rate, accelerated breathing, and abdominal swelling. At 24 h after CLP, the rats trembled, had pus in their eyes, and exhibited a tendency to bleed. The viscera of the rats were observed by laparotomy. A massive, bloody, and turbid exudate with a foul odor was observed in the abdominal cavity of the rats in the CLP and CLP+Cit groups. Notable inflation of the small intestine, blackening of the ligated section of the ceca, necrosis and adhesions, evident edema in the liver, and different degrees of circular bleeding points on the surface of the liver and kidneys were observed. The rats in the normal group, sham group, and Cit group did not exhibit evident abnormalities. Comparing the CLP group and the CLP+Cit group, the general condition of the two groups had little difference. But the intra-abdominal lesions of the CLP group were more serious than in the CLP+Cit group. At 24h after CLP, rats in the CLP group showed large amounts of bloody ascites and severe adhesion in the surrounding viscera of the cecum. At the same time point, the CLP+Cit group had less ascites and the ascites was more clear. The necrosis of the intestine and visceral adhesion in the CLP+Cit group were also lighter than those in the CLP group.

### 3.3. Pathology of Rat Heart Tissues

**Figure 1:**
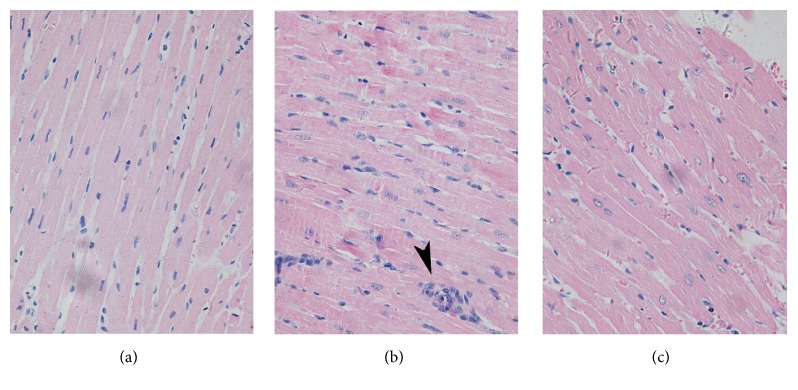
Pathology of rat heart tissues. (a) The striated myocardial fibers of rats in the normal groups exhibited clear structures. The nuclei of the muscle fibers were ovoid and were located in the center of the cell. (b) The myocardial fibers of rats in the CLP 24 h group were infiltrated by inflammatory cells, as indicated by the arrow. (c) In the rats of the CLP+Cit 24h group, inflammatory cell infiltration around the myocardial fibers was milder than that in the rats of the CLP 24h group. There was little change in the size of the cardiac myocytes, and exudation was visible around the myocardial cells.

Using light microscopy (×400) and HE staining, pathological changes were observed in the striated myocardial fibers (Figure 1). The striated myocardial fibers of rats in the normal groups exhibited clear structures. The nuclei of the muscle fibers were ovoid and were located in the center of the cell. The myocardial fibers of rats in the CLP 24h group were infiltrated by inflammatory cells. In the rats of the CLP+Cit 24h group, inflammatory cell infiltration around the myocardial fibers was milder than that in the rats of the CLP 24h group. In the CLP+Cit 24h group, there was little change in the size of the cardiac myocytes, and exudation was visible around the myocardial cells.

### 3.4. Routine Blood Tests and Serum Tests

#### 3.4.1. Blood Cell Count

As shown in Table 1, the neutrophil percentage and absolute neutrophil count were both higher in the rats of the sham group than in the rats of the normal group and Cit group (all* P*<0.05). Neutrophil percentage and absolute neutrophil count of rats in the CLP and CLP+Cit groups increased with time. At the three time points of 6 h, 12 h, and 24 h after CLP surgery, the neutrophil percentage and absolute neutrophil count were both lower for rats in the CLP+Cit group than for rats in the CLP group, and the differences were statistically significant (all* P*<0.05), except for the neutrophil percentage in the 6-h CLP subgroup (*P*>0.05). The leukocyte count and platelet count in the rats of the sham and Cit groups were higher than those in the rats of the normal control group; however, the differences were not statistically significant (all* P*>0.05). The leukocyte count of rats in the CLP and CLP+Cit groups increased with time. At the three time points of 6 h, 12 h, and 24 h after CLP surgery, the leukocyte count was lower for rats in the CLP+Cit group than for rats in the CLP group, and the differences were statistically significant (all* P*<0.05). And the platelet count in the CLP and CLP+Cit groups was lower than that in the normal group and much lower than that in the sham group. At the three time points of 6 h, 12 h, and 24 h after CLP surgery, the platelet count was higher in the rats of the CLP+Cit group than in the rats of the CLP group; however, there was no statistically significant difference (all* P*>0.05). There were no statistically significant differences in the hemoglobin concentration and erythrocyte count between any two groups of rats (all* P*>0.05).

#### 3.4.2. Serum Myocardial Enzymes

The levels of CK, CK-MB, LDH, and HBDH in the normal group, sham group, and Cit group were not significantly different (*P*>0.05). The CLP and CLP+Cit groups were compared at the three time points of 6 h, 12 h, and 24 h after CLP surgery, and the results are shown in Table 2. No significant differences were observed for LDH and CK-MB in the 12-h and 24-h subgroups (*P*>0.05). CK in the CLP+Cit group was significantly lower in the 6 h and 24h subgroups than that in the CLP group (*P*<0.05). And the CK 12 h subgroup, CK-MB 6h subgroup, and HBDH 6h, 12h, and 24h subgroups in the CLP+Cit group were more significantly lower than those in the CLP group (*P*<0.01).

### 3.5. NO, iNOS, SOD, and MDA Determination

**Figure 2:**
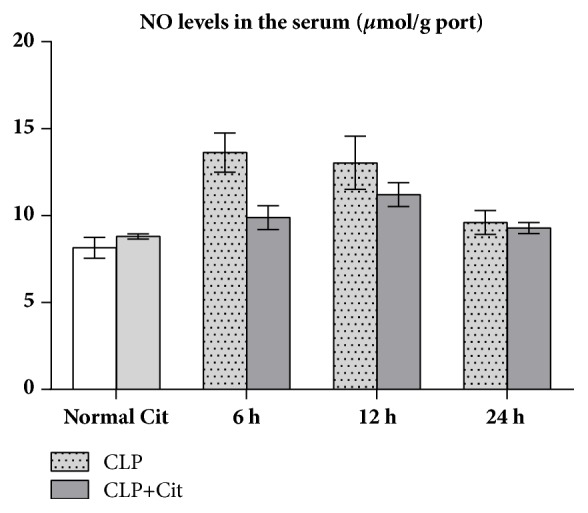
In the 6-h and 12-h subgroups, NO levels were significantly higher in the CLP group than in the CLP+Cit group (*P*<0.05).

**Figure 3:**
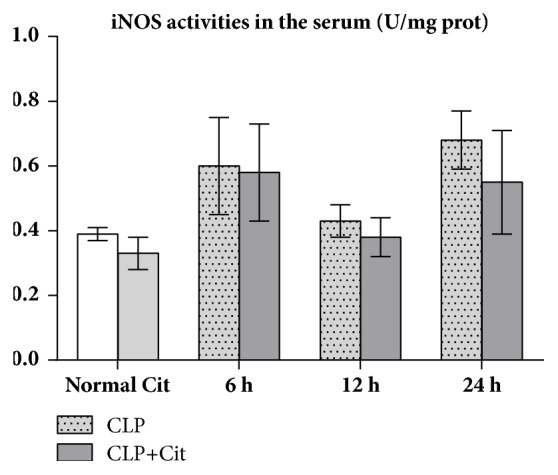
In the 6-h, 12-h, and 24-h subgroups, iNOS activity was higher in the CLP group than in the CLP+Cit group; however, the difference was not statistically significant (all* P*>0.05).

As shown in Table 3, the serum NO levels and iNOS activity were not significantly different between the normal and Cit groups (all* P*>0.05). Within 24 h after CLP surgery, the NO levels and iNOS activity in the CLP and CLP+Cit groups were both higher than those in the normal group. At 6 h and 12 h but not 24 h after CLP, NO levels in the CLP group were significantly higher than those in the CLP+Cit group (*P*<0.05) (Figure 2). In addition, at the three time points, the iNOS activity in the CLP group was higher than that in the CLP+Cit group; however, the difference was not statistically significant (all* P*>0.05) (Figure 3).

**Figure 4:**
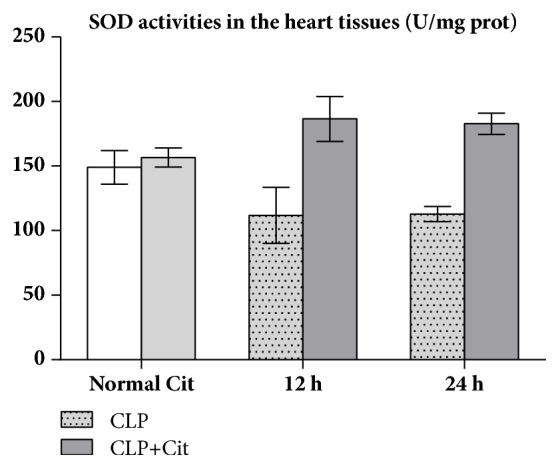
In the 24-h and 12-h subgroups, SOD activity was significantly higher in the CLP+Cit group than in the CLP group (*P*<0.05).

**Figure 5:**
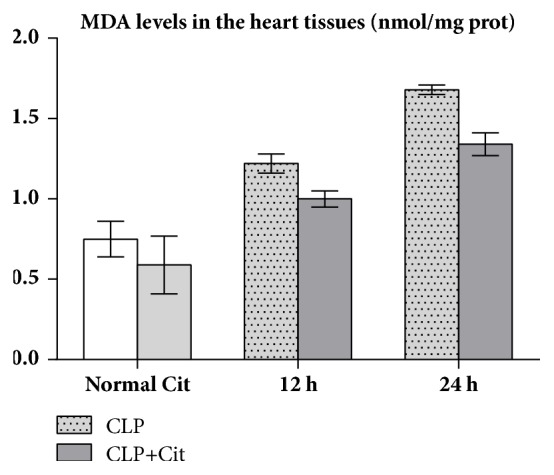
In the 12-h and 24-h subgroups, MDA levels were significantly higher in the CLP group than in the CLP+Cit group (*P*<0.05).

As shown in Table 4, the SOD activity and the MDA levels in the heart tissues did not differ significantly between the normal group and the Cit group (all* P*>0.05). In the 24-h and 12-h subgroups, the SOD activity of the CLP+Cit group was higher than that of the CLP group (*P*<0.05) (Figure 4), and the MDA levels in the CLP group were higher than those in the CLP+Cit group (*P*<0.05) (Figure 5).

## 4. Discussion

### 4.1. Effects of Cit on Infection in Septic Rats

Infection is the main cause of sepsis, as it leads to the excessive release of proinflammatory mediators and increases the inflammatory response time. Additionally, a large number of inflammatory mediators with immunosuppressive effects are released, which leads to decreased immunity and promotes the development of MODS [17]. The numbers of leukocytes, neutrophils, and platelets in deceased patients with sepsis were significantly lower than those in survivors [18]. In addition, the extent of the platelet count reduction was positively correlated with the severity of sepsis [19]. Thus, platelet count reduction can be used as an effective early warning index of the severity and prognosis of septic patients. We found that, in a previous study, exogenous Cit alleviated the release of proinflammatory cytokines (TNF-*α*, IL-1*β*, and IL-6) in early sepsis and increased the release of late-stage anti-inflammatory cytokines (IL-4 and IL-10) to alleviate the inflammatory response of septic rats [4].

In our study, the leukocyte count, the absolute neutrophil count, and the neutrophil percentage of the CLP and CLP+Cit groups increased with time and were higher than those of the normal group. However, the platelet count in the CLP and CLP+Cit groups was lower than that in the normal group and much lower than that in the sham group. Altogether, these results indicate that, after the CLP model was established, rats exhibited an inflammatory response of varying degree, an increase in inflammatory cells, and a decreased platelet count. Moreover, at the three time points of 6 h, 12 h, and 24 h after CLP surgery, the leukocyte count, the absolute neutrophil count, and the neutrophil percentage in the CLP+Cit group were all less than those in the CLP group, indicating that the addition of exogenous Cit can effectively control the inflammatory response and reduce inflammatory cell activation and the release of inflammatory mediators.

### 4.2. Effect of Cit on Cardiac Function in Septic Rats

Organ dysfunction in sepsis involves multiple mechanisms, such as changes in organ microenvironment, inflammatory factor effects, oxidative stress injury, mitochondrial dysfunction, and tissue cell apoptosis. Studies have shown that supplementation of appropriate amounts of ARG can reduce intestinal mucosal epithelial cell apoptosis in rats with severe abdominal infection after CLP and improve the prognosis of infected rats [20]. During sepsis, the production of endogenous ARG is approximately 30% of normal [21]. However, supplementation with a large amount of ARG leads to overproduction of NO and induces more inflammatory factors and, thus, cell damage. As an ARG precursor, Cit can promote the synthesis of ARG, improve the action of ARG, and provide negative feedback to regulate the formation of NO [22]. Studies have shown that Cit has protective effects against ischemia-reperfusion injury of organs via inhibition of excessive NO production [23]. When cells of organ tissues are damaged and undergo apoptosis, the changes in organ function can be examined by evaluating the related indexes. CK, CK-MB, LDH, and *α*-HBDH are common myocardial enzymes that reflect the extent of myocardial injury. CK is mainly distributed in skeletal muscle, followed by the myocardium. Its isoenzymes include CK-BB, CK-MB, CK-MM, and CK-Mt, and CK-MB is mainly expressed in the cytoplasm of the myocardium [24]. The isoenzymes of LDH include LDH1, LDH2, LDH3, LDH4, and LDH5, and the level of LDH1 in the myocardium is higher than the level of the other isoenzymes [25]. Finally, HBDH uses *α*-HBDH as a substrate, which reflects the sum of LDH1 and LDH2 activities [26].

In our study, visualization under a light microscope revealed that the myocardial fibers of CLP rats were infiltrated by inflammatory cells and that the myocardial cells were thinner. Additionally, marked myocardial interstitial edema was observed after HE staining. Rats in the CLP+Cit group exhibited milder inflammatory cell infiltration around cardiac muscle fibers than rats in the CLP group. There was little change in the size of the cardiac myocytes, and exudation was visible around the myocardial cells. Blood biochemical detection of myocardial enzymes showed that the levels of CK, CK-MB, LDH, and HBDH in the CLP and CLP+Cit groups were higher than those in the normal group, revealing that the myocardial cells in the CLP and CLP+Cit groups were damaged due to inflammation after the CLP model was established. At the three time points of 6 h, 12 h, and 24 h after CLP surgery, the serum levels of CK, LDH, and HBDH in the CLP+Cit group were all lower than those in the CLP group, revealing that supplementation of exogenous Cit could reduce inflammation in myocardial cells during early sepsis and alleviate myocardial injury.

### 4.3. Oxidative Stress and the Effects of NO

The synthesis and release of cytokines increase during sepsis. A large number of cytokines that act on cardiomyocytes can reduce the responsiveness of beta-adrenergic receptors and thus affect myocardial systolic function [27]. Additionally, myocardial ischemia can cause myocardial injury. Myocardial contraction disorder leads to a decrease in peripheral blood flow, thereby causing ischemia of the tissues. Moreover, the increase in oxygen free radicals and tissue damage are considered the main causes of multiple-organ dysfunction related to sepsis [28]. The human body has an oxidation system and an antioxidation system. Reactive oxygen species (ROS) constitute the main oxidation system, including O_2_^−^, OH, and H_2_O_2_, whereas the antioxidant system includes SOD and GSH-PX. Under normal conditions, both the oxidation system and the antioxidation system are in balance [29]. However, when the antioxidant molecules in cells (glutathione, vitamin E, etc.) cannot process large amounts of accumulated ROS in a timely manner, ROS and a specific nitric oxide synthase that is present in the mitochondria produce more NO under hypoxic conditions. NO reacts with superoxide anion (O_2_^−^) to generate peroxynitrite (ONOO^−^), which can cause irreversible oxidative damage to the tyrosine residue of proteins, resulting in tissue cell damage. In addition, large numbers of neutrophils lead to an increase in oxygen consumption during phagocytosis. The ingested oxygen is catalyzed by intracellular NADPH oxidase and NADH oxidase, which accept electrons to generate large amounts of oxygen free radicals. When the scavenging of oxygen free radicals is insufficient or the level of antioxidants is insufficient and oxygen free radicals are produced to kill pathogens, damage can also occur in the organism [30]. In response to endotoxins and inflammatory cytokines, iNOS activity increases and induces the formation of more NO. In the process of converting Arg to Cit, more NO is produced, which aggravates the inflammatory response [31]. Many experiments have shown that antioxidative therapy has a significant effect on patients with sepsis. Detection of antioxidant enzymes is one of the most common methods for detecting oxidative stress injury in cells. Among the enzymes, SOD is a type of acidic metal protein that mainly exists in the cytoplasm and mitochondria of eukaryotic cells. SOD can remove the superoxide anion (O_2_^−^) and protect cells from damage [32].

In our study, the activity of SOD in the heart tissues of the CLP group was lower than that in the heart tissues of the normal group, which indicated that the antioxidative ability of visceral cells decreased after CLP. The SOD activity in heart tissue was higher in the CLP+Cit group than in the CLP group, which indicates that exogenous Cit could improve the antioxidant ability of the hearts of septic rats. MDA is a common index used to reflect the degree of lipid peroxidation in the body, which can indirectly reflect the extent of cell damage. The level of MDA in cardiac tissues of the CLP+Cit and CLP groups was higher than that of the normal group, which indicated that the cytomembrane of heart tissue cells was damaged after CLP. The level of MDA in cardiac tissue of the CLP+Cit group was significantly lower than that of the CLP group, suggesting that exogenous Cit could reduce lipid peroxidation-induced damage to the cytomembrane of heart tissue cells. Regarding reactive nitrogen species (RNS), under normal conditions, NO is produced by nitric oxide synthases (NOSs), which catalyze ARG production. Under pathological conditions of sepsis, a high concentration of NO can activate the NF-*κ*B signaling pathway and induce proinflammatory cytokines (TNF-*α*, IL-1*β*, and others). IL-1 and TNF-*α* also activate iNOS and promote the production of more NO, which results in a positive feedback loop that causes prolonged and increased acute inflammation, thereby damaging the organism. Within 24 h after the CLP operation, the serum NO level and iNOS activity in the CLP rats were higher than those in normal rats, which indicates that, under inflammatory conditions, iNOS activity in the blood is increased, resulting in increased NO production. At the 6-h and 12-h time points, the serum NO concentration in the CLP+Cit group was significantly lower than that in the CLP group, indicating that exogenous Cit could reduce NO production in the blood and alleviate the damage caused by inflammation to the organism. The generation of NO is related to iNOS activity. However, although serum iNOS activity in the rats was notably lower in the CLP+Cit group than in the CLP group at the three time points, the differences between the two groups were not statistically significant.

## 5. Conclusion

Based on our experimental results, we can draw the following conclusions:

(1) Supplementation of exogenous citrulline can improve the 24 h survival rate and reduce inflammation in the abdominal cavity of septic rats

(2) Supplementation of exogenous citrulline can effectively control the inflammatory response and reduce inflammatory cell activation and the release of inflammatory mediators

(3) Supplementation of exogenous citrulline can reduce inflammatory cell infiltration around the myocardial cells, reduce inflammation in myocardial cells during early sepsis, and alleviate myocardial injury in septic rats

(4) Supplementation of exogenous citrulline can increase the antioxidant capacity of the heart and reduce the damage of lipid peroxidation on cardiac cell membrane in septic rats

Therefore, we have reason to believe that exogenous citrulline supplementation can relieve sepsis in septic rats. We discussed some of the mechanisms based on the relevant detection indicators in Discussion, and more mechanisms need to be further explored.

## Figures and Tables

**Table 1:** Neutrophil percentage, absolute neutrophil count, leukocyte count, platelet count, hemoglobin concentration, and erythrocyte count of rats (x±s) **(**^**∗**^**P<0.05 and **^**∗∗**^**P<0.05).**

**Group**	Neutrophil percentage(%)			Absolute neutrophils count **(10**^**9**^**/L)**			Leukocytes **(10**^**9**^**/L)**			Platelets **(10**^**9**^**/L)**			Hemoglobin **(g/L)**			Erythrocytes **(10**^**12**^**/L)**		
	**6 h**	**12 h**	**24 h**	**6 h**	**12 h**	**24 h**	**6 h**	**12 h**	**24 h**	**6 h**	**12 h**	**24 h**	**6 h**	**12 h**	**24 h**	**6 h**	**12 h**	**24 h**
**Normal**	0.05 ± 0.01			0.33 ± 0.37			3.53 ± 1.15			730 ± 45.57			144.00 ± 2.65			6.70 ± 0.13		
**Sham**	0.44 ± 0.08			1.52 ± 0.36			4.84 ± 0.47			834.73 ± 62.33			136.83 ± 3.42			6.07 ± 0.19		
**Cit**	0.10 ± 0.09			0.53 ± 0.27			5.28 ± 0.83			778.00 ± 144.90			139.00 ± 9.14			6.04 ± 0.45		
**CLP**	0.24 ± 0.07	0.44 ± 0.01	0.56 ± 0.03	1.66 ± 0.24	2.07 ± 0.41	4.25 ± 0.31	5.06 ± 0.27	4.53 ± 0.72	6.85 ± 1.30	555.25 ± 219.85	434.00 ± 39.50	433.67 ± 74.20	149.33 ± 15.89	129.33 ± 25.24	157.50 ± 5.51	6.56 ± 0.80	5.47 ± 1.80	7.63 ± 1.28
**CLP+Cit**	0.19 ± 0.02	0.12 ± 0.07*∗∗*	0.10 ± 0.10*∗∗*	0.62 ± 0.12*∗*	0.20 ± 0.14*∗∗*	0.29 ± 0.12*∗∗*	3.58 ± 0.79*∗*	2.33 ± 0.44*∗*	2.10 ± 0.50*∗∗*	688.85 ± 178.84	525.20 ± 64.76	511.50 ± 46.87	149.26 ± 10.87	151.75 ± 9.61	184.25 ± 24.91	6.84 ± 0.58	6.91 ± 0.66	8.63 ± 1.13

**Table 2:** Levels of CK, CK-MB, LDH, and HBDH (x±s).

	**CK (U/L)**			**CK-MB (U/L)**			**LDH (U/L)**			**HBDH (U/L)**		
**Group**	**6 h**	**12 h**	**24 h**	**6 h**	**12 h**	**24 h**	**6 h**	**12 h**	**24 h**	**6 h**	**12 h**	**24 h**
**Normal**	1104.6±25.83			1169.8±259.2			1373.5±482.6			111.2±93.78		
**Sham**	1081.0±67.13			2312.3±665.6			1637.75±233			1137.3±77.05		
**Cit**	1085.8±48.66			2590±156.42			1818±185.98			1081.3±26.25		
**CLP**	6505.67±129.14	4421.5±522.9	2694.5±194.1	3511.5±166.1	2514.2±517.5	1615.5±278.5	2774.3±150.2	2577±273.15	1417.3±99.6	1791±151.65	2418±232.46	938.5±62.4
**CLP+Cit**	4446.3±475.59**∗**	1309±89.3**∗∗**	2133.3±322.9**∗**	22067±73.1**∗∗**	2639±109.66	1819.7±456.1	2604.8±256.3	2076±309.36	1595.67±222.8	1562±38.08**∗∗**	1126±13.65**∗∗**	696.7±57.6**∗∗**

^*∗*^ *P*<0.05 and ^*∗∗*^*P* <0.01.

**Table 3:** Serum NO levels and iNOS activity (x±s).

**Group**	**NO (** ***μ*** **mol/g prot)**			**iNOS (U/mg prot)**		
	**6 h**	**12 h**	**24 h**	**6 h**	**12 h**	**24 h**
**Normal**	8.15±0.60			0.39±0.02		
**Cit**	8.79±0.15			0.33±0.05		
**CLP**	13.62±1.13	13.03±1.53	9.60±0.68	0.60±0.15	0.43±0.05	0.68±0.09
**CLP+Cit**	9.88±0.68*∗∗*	11.20±0.68*∗*	9.28±0.32	0.58±0.15	0.38±0.06	0.55±0.16

^*∗*^ *P* <0.05 and ^*∗∗*^*P* <0.01.

**Table 4:** SOD activity and MDA levels in the heart tissues (x±s).

**Group**	**SOD (U/mg prot)**		**MDA (nmol/mg prot)**	
	**12 h**	**24 h**	**12 h**	**24 h**
**Normal**	149.03±13.00		0.75±0.11	
**Cit**	156.65±7.44		0.59±0.18	
**CLP**	111.71±21.63	112.77±5.87	1.22±0.06	1.68±0.03
**CLP+Cit**	186.46±17.42**∗**	182.75±8.27**∗∗**	1.00±0.05**∗∗**	1.34±0.07**∗**

^*∗*^ *P* <0.05 and ^*∗∗*^*P* <0.01.

## Abbreviations

Cit: Citrulline
CLP: Cecal ligation and puncture
CK: Creatine kinase
CK-MB: Creatine kinase isoenzyme
LDH: Lactate dehydrogenase
HBDH: *α*-Hydroxybutyrate dehydrogenase
iNOS: Inducible nitric oxide synthase
SOD: Superoxide dismutase
MDA: Malondialdehyde
HE: Hematoxylin-eosin
SIRS: Systemic inflammatory response syndrome
MODS: Multiple-organ dysfunction syndrome
SOFA: Sepsis-related Organ Failure Assessment
NADPH: Nicotinamide adenine dinucleotide phosphate
LPS: Lipopolysaccharide
ARG: Arginine
SPF: Specified pathogen-free
SD: Standard deviation
RNS: Reactive nitrogen species.
